# Anti-N-Methyl-D-Aspartate Receptor Encephalitis With Autonomic Instability and Focal Seizures Without Detectable Ovarian Teratoma

**DOI:** 10.7759/cureus.118052

**Published:** 2026-10-17

**Authors:** Pavithra Muthiah, Mohiuddin Sharief

**Affiliations:** 1 Acute Medicine, Lancashire Teaching Hospitals NHS Foundation Trust, Preston, GBR

**Keywords:** anti-nmda receptor encephalitis, autoimmune encephalitis, autonomic dysfunction, encephalitis, focal seizures, rituximab, sinus tachycardia

## Abstract

Anti-N-methyl-D-aspartate receptor (anti-NMDAR) encephalitis is a severe but potentially reversible form of autoimmune encephalitis, and it typically affects young women. It can be challenging to differentiate from other conditions that cause symptoms such as seizures and psychiatric and autonomic manifestations. We present the case of a previously healthy young woman without any significant past medical history who developed acute-onset, progressive neuropsychiatric disturbance, focal impaired awareness seizures, cognitive impairment, and intermittent autonomic disturbance manifesting as sinus tachycardia. Initial investigations showed mild pleocytosis in the CSF and subtle hyperintensity involving the right insular region and adjacent subcortical white matter on MRI. Further tests detected anti-NMDAR antibodies in both serum and CSF. She was started on first-line treatment with IV methylprednisolone and IVIG, but persistent cognitive impairment prompted escalation to second-line treatment with rituximab. She also underwent an extensive workup for an underlying ovarian teratoma or other neoplasms, including pelvic ultrasound and MRI, both of which were unremarkable. The patient eventually experienced gradual neurological recovery with immunotherapy and neurorehabilitation, ultimately regaining her independence and returning home. This case highlights autonomic dysfunction as a possible feature of anti-NMDAR encephalitis and the importance of early immunotherapy, even in patients without an identifiable tumor.

## Introduction

Anti-N-methyl-D-aspartate receptor (anti-NMDAR) encephalitis is among the most common forms of autoimmune encephalitis and predominantly affects young women [1,2]. The disease typically evolves over days to weeks, presenting with a constellation of neuropsychiatric symptoms, including behavioral and personality changes, memory impairment, seizures, movement disorders, autonomic dysfunction, and altered levels of consciousness [2,3]. Although ovarian teratomas are classically associated with anti-NMDAR encephalitis, a substantial proportion of patients have no identifiable underlying tumor.

Autonomic dysfunction is a recognized feature of anti-NMDAR encephalitis, particularly in more severe cases [3]; however, it may be overlooked during the early stages of the disease. This can prompt extensive cardiac investigations, potentially delaying recognition of the underlying neurological disorder. Awareness of these atypical presenting features is therefore essential to facilitate timely diagnosis and initiation of immunotherapy [1,2].

We describe the case of a young woman with anti-NMDAR encephalitis who presented with recurrent marked sinus tachycardia, focal seizures, right-predominant electroencephalographic slowing, and MRI abnormalities involving the right insular region and adjacent subcortical white matter. The insular cortex forms an important component of the central autonomic network and has been implicated in cardiovascular regulation and autonomic imbalance. Despite the absence of an identifiable ovarian teratoma, the patient required escalation from first-line immunotherapy with corticosteroids and IVIG to second-line treatment with rituximab because of persistent cognitive deficits [4-6]. This case highlights the diagnostic challenges posed by autonomic manifestations of autoimmune encephalitis and the importance of early recognition and timely immunotherapy, while raising the possibility of a relationship between autonomic dysfunction and focal imaging abnormalities, although causality cannot be established from a single observation.

## Case presentation

A previously healthy woman in her mid-20s presented with a two- to three-week history of progressively worsening neurological and psychiatric symptoms. Her illness initially began with abnormal oral sensations involving the lips and tongue, followed by intermittent slurred speech. Over the following days, she developed recurrent episodes of impaired awareness associated with staring, lip smacking, facial automatisms, and unilateral limb movements. Her family also reported marked behavioral changes, including increasing paranoia, emotional lability, and visual and auditory hallucinations. She additionally expressed thoughts of self-harm.

On admission, she was intermittently confused and displayed impaired short-term memory. Neurological examination revealed fluctuating facial twitching, hyperreflexia, increased muscle tone, and mild unilateral weakness. During her hospital stay, she experienced multiple focal impaired awareness seizures accompanied by orofacial automatisms.

A notable feature of her presentation was recurrent autonomic instability. Continuous telemetry monitoring demonstrated repeated episodes of narrow-complex sinus tachycardia, with heart rates of 170-180 beats/min. These episodes prompted cardiology review, transthoracic echocardiography, serial electrocardiograms, troponin testing, and assessment for pulmonary embolism. The electrocardiogram demonstrated sinus tachycardia without other arrhythmias (Figure 1). Echocardiography showed preserved biventricular function without structural abnormalities and was otherwise unremarkable. Ventilation-perfusion scanning showed normal pulmonary perfusion and no evidence of pulmonary embolism. The tachycardia was managed supportively with a low-dose beta-blocker.

**Figure 1:**
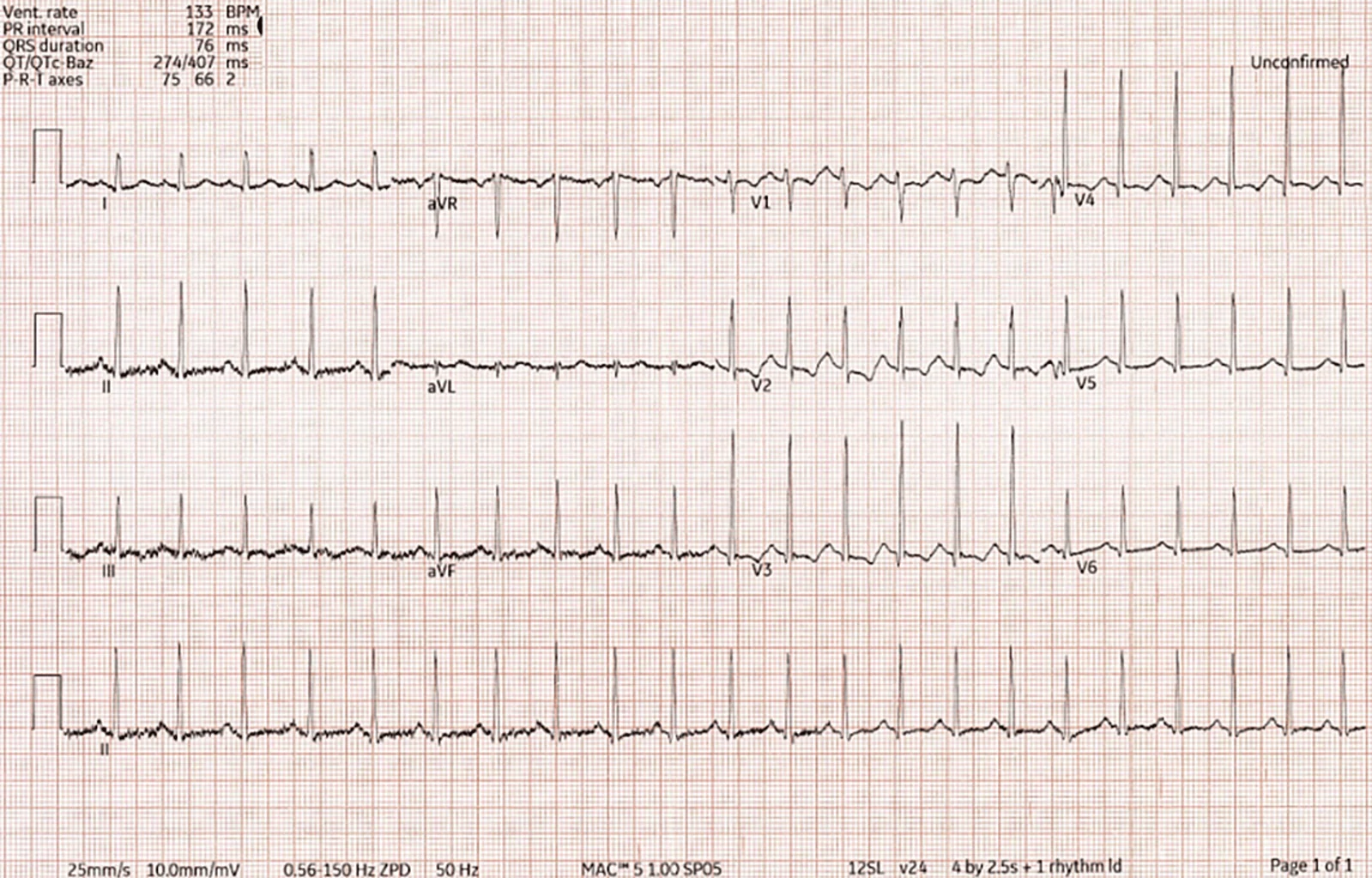
Twelve-lead electrocardiogram demonstrating sinus tachycardia during an episode of autonomic instability.

Initial laboratory investigations revealed no significant inflammatory response. CSF analysis demonstrated mild pleocytosis, with a white blood cell count of 26 × 10⁶/L (normal range <5 × 10⁶/L), but a normal protein concentration and negative bacterial cultures. Anti-NMDAR antibodies (cell-based assay) were detected in both serum and CSF, while other antibodies were negative. Additional CSF studies demonstrated oligoclonal IgG bands, indicating intrathecal immunoglobulin synthesis, and testing for herpes simplex virus types 1 and 2, varicella-zoster virus, enterovirus, and parechovirus was negative. Treponemal serology and Lyme disease screening were also negative. These findings are summarized in Table 1.

**Table 1:** Summary of laboratory and CSF investigations. * Reference ranges may vary between laboratories. HSV, herpes simplex virus; NMDAR, N-methyl-D-aspartate receptor; VZV, varicella-zoster virus

Investigation	Result	Reference range	Interpretation
CSF white cell count	26 × 10⁶/L	<5 × 10⁶/L*	Mild pleocytosis
CSF lymphocytes	80%	N/A	Lymphocytic predominance
CSF protein	Normal	0.15-0.45 g/L*	Normal
CSF culture	No growth	Negative	No bacterial infection
HSV-1 PCR	Negative	Negative	No evidence of HSV infection
HSV-2 PCR	Negative	Negative	No evidence of HSV infection
VZV PCR	Negative	Negative	No evidence of VZV infection
Enterovirus PCR	Negative	Negative	No evidence of enterovirus infection
Parechovirus PCR	Negative	Negative	No evidence of parechovirus infection
Anti-NMDAR antibodies	Positive (serum and CSF)	Negative	Diagnostic of anti-NMDAR encephalitis
LGI1 antibodies	Negative	Negative	LGI1 antibodies not detected
CASPR2 antibodies	Negative	Negative	CASPR2 antibodies not detected
Oligoclonal bands	Present in CSF	Absent	Intrathecal IgG synthesis
CSF IgG	0.027 g/L	0.00-0.034 g/L*	Within range
CSF albumin	0.16 g/L	0.10-0.35 g/L*	Within range
CSF IgG/albumin ratio	0.17	<0.21*	Normal barrier function
D-dimer	1.57 mg/L	<0.50 mg/L	Elevated
Homocysteine	11 μmol/L	5-15 μmol/L*	Normal
Treponemal serology	Negative	Negative	No evidence of syphilis
Lyme serology	Negative	Negative	No evidence of Lyme disease

CT of the head was unremarkable. MRI of the brain demonstrated T2-weighted and fluid-attenuated inversion recovery (FLAIR) hyperintensity within the right insular cortex, extending into the adjacent subcortical white matter, interpreted as either seizure-related changes or encephalitic involvement (Figures 2, 3). MRI of the spine was normal.

**Figure 2:**
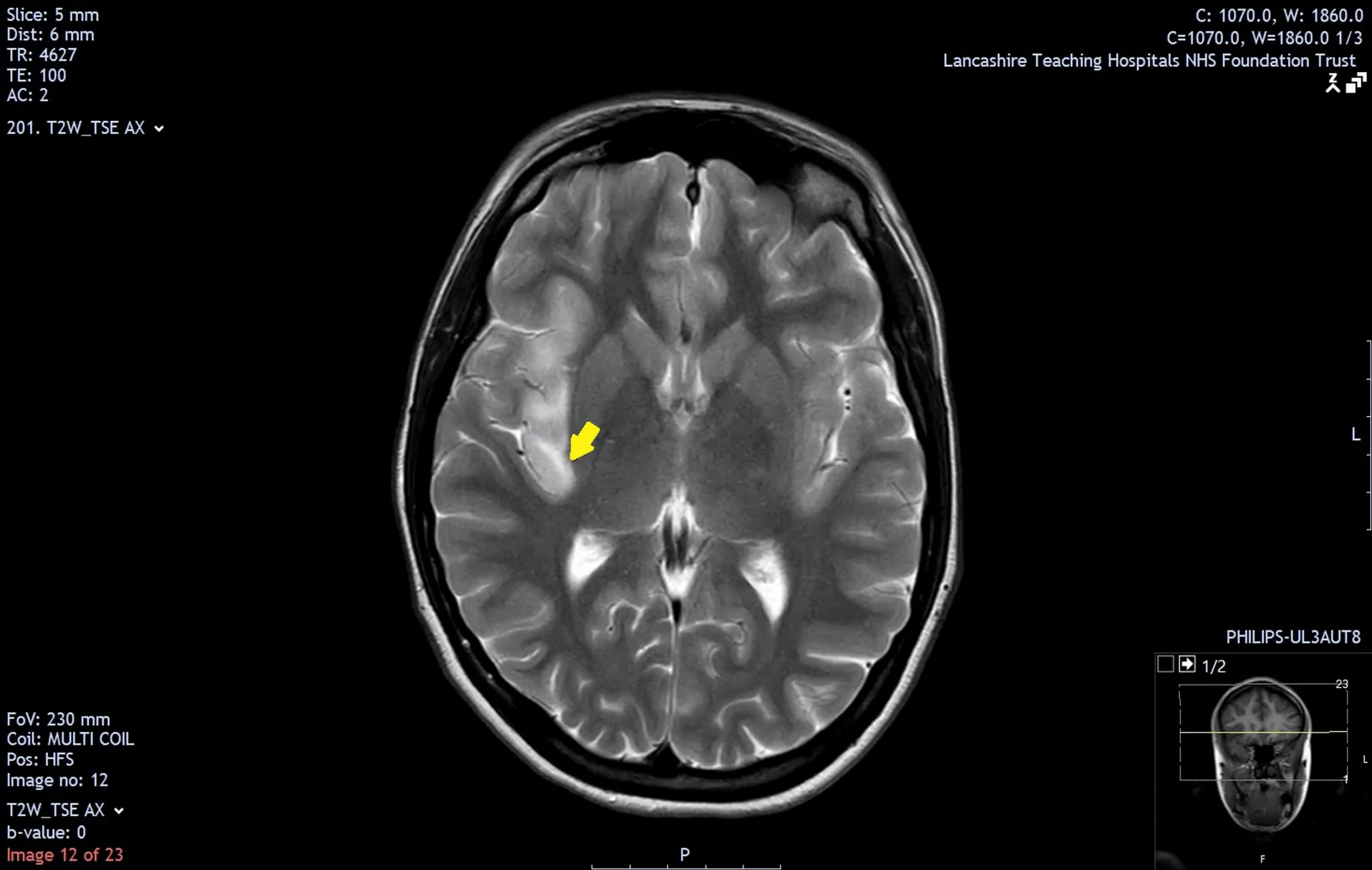
Axial T2-weighted MRI demonstrating focal hyperintensity involving the right insular region and adjacent subcortical white matter (yellow arrow).

**Figure 3:**
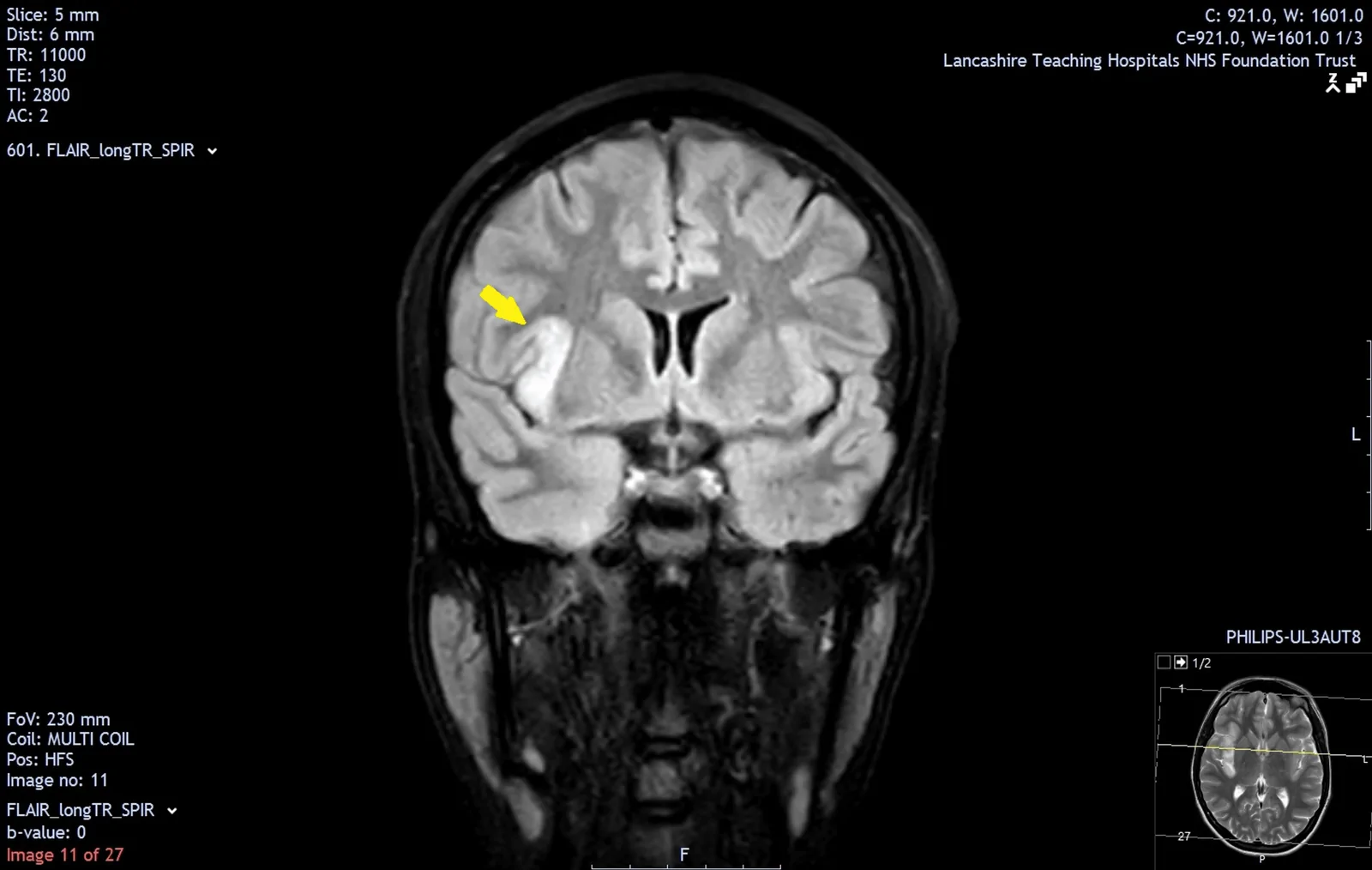
Coronal FLAIR MRI demonstrating corresponding hyperintensity involving the right insular region and adjacent subcortical white matter (yellow arrow), compatible with inflammatory or seizure-related changes. FLAIR, fluid-attenuated inversion recovery

Electroencephalography (EEG) demonstrated a poorly sustained posterior dominant rhythm at 6-8 Hz (amplitude up to 40 μV), with frequent right-predominant delta activity at 1.5-3.5 Hz (amplitude up to 55 μV), consistent with encephalopathy (Figure 4). No epileptiform discharges, electrographic seizures, or extreme delta brush pattern were observed.

**Figure 4:**
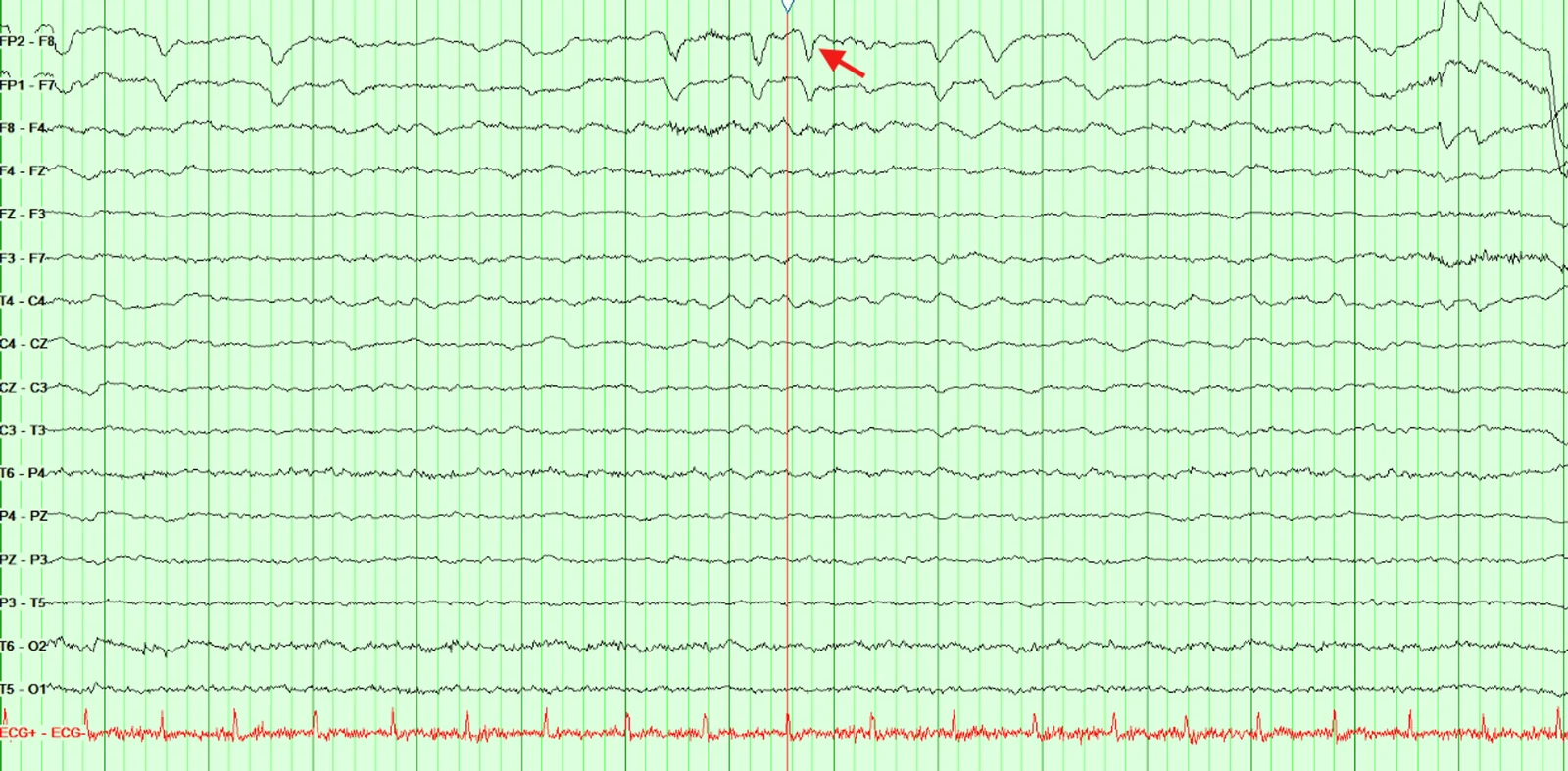
Electroencephalogram demonstrating right-predominant delta slowing. The red arrow highlights semirhythmic delta activity over the right hemisphere, consistent with encephalopathy. No epileptiform discharges or electrographic seizures were identified.

Empirical treatment for infectious encephalitis was initiated with IV aciclovir and ceftriaxone while further investigations were undertaken. Given the combination of new-onset focal seizures, psychiatric symptoms, cognitive impairment, autonomic instability, and CSF pleocytosis, autoimmune encephalitis was strongly suspected.

Given the clinical suspicion of autoimmune encephalitis, the patient received IV methylprednisolone at 1 g daily for five days, followed by oral prednisolone at 60 mg daily with a gradual taper. Gastroprotection and bone protection were provided with lansoprazole and calcium/vitamin D supplementation. Persistent neurological and cognitive deficits prompted treatment with IVIG. Subsequently, anti-NMDAR antibodies returned positive in both serum and CSF, confirming the diagnosis of anti-NMDAR encephalitis. Antiseizure therapy consisted of levetiracetam at 1,500 mg and lacosamide at 150 mg. Intermittent lorazepam was administered as required for breakthrough symptoms. Recurrent sinus tachycardia was managed with low-dose bisoprolol.

Given the known association between anti-NMDAR encephalitis and underlying neoplasms, particularly ovarian teratomas, the patient underwent further investigations. Pelvic ultrasonography and pelvic MRI demonstrated normal ovaries and uterus, without evidence of a teratoma or other pelvic pathology (Figure 5A, 5B).

**Figure 5:**
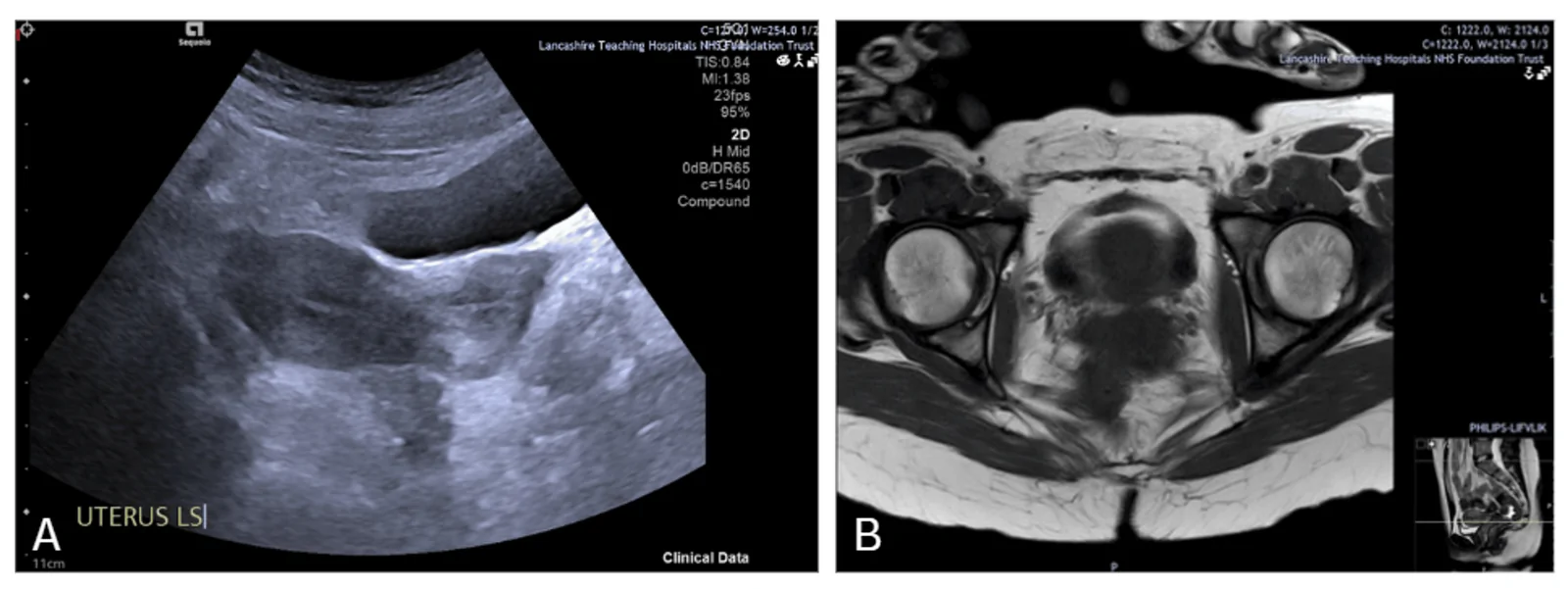
Pelvic ultrasound and MRI demonstrating no evidence of ovarian teratoma. (A) Pelvic ultrasound demonstrating no sonographic evidence of an adnexal mass. (B) Axial T2-weighted pelvic MRI confirming the absence of an ovarian teratoma or other pelvic pathology. No underlying neoplastic trigger was identified despite comprehensive pelvic imaging.

Despite gradual improvement following first-line immunotherapy, the patient continued to exhibit significant cognitive deficits and memory impairment. Following multidisciplinary discussion, treatment was escalated to rituximab [7]. She received two rituximab infusions two weeks apart. The first infusion was complicated by transient chest tightness and dyspnea, which resolved after temporarily interrupting the infusion and restarting it at a slower rate.

Over the following weeks, the patient demonstrated substantial neurological recovery. Cognitive function, insight, and verbal fluency progressively improved. During recovery, she experienced brief episodes of eyelid fluttering that remained distractible, with preserved awareness, and were considered functional nonepileptic events rather than epileptic seizures.

She participated actively in physiotherapy, occupational therapy, and neurorehabilitation. By discharge, she was independently mobile, seizure-free, cognitively improved, and able to undertake community-based rehabilitation with ongoing neurological follow-up. A detailed summary of the patient’s clinical course is shown in Table 2.

**Table 2:** Clinical timeline demonstrating symptom progression, diagnostic investigations, immunotherapy, rituximab escalation, and neurological recovery. NMDAR, N-methyl-D-aspartate receptor

Time point	Clinical event
Initial presentation	Onset of oral sensory symptoms and speech disturbance
Symptom progression	Development of focal impaired awareness seizures
Hospital presentation	Behavioral change, hallucinations, paranoia, hospital admission
Admission day 0	Ongoing psychiatric symptoms, seizures, cognitive impairment, and dysautonomia. MRI and CSF investigations performed. Recurrent sinus tachycardia at 170-180 bpm requiring telemetry monitoring and cardiology review.
Admission day 3	IV methylprednisolone initiated
Admission week 2	Anti-NMDAR antibodies confirmed in serum and CSF
Admission week 2 onwards	IVIG administered. Given oral prednisolone. Continued immunotherapy and antiseizure medications.
Weeks 5-8	Rituximab administered in two doses, two weeks apart, due to persistent cognitive dysfunction
Discharge week 12	Improved cognition, seizure-free, independent mobility

## Discussion

Anti-NMDAR encephalitis commonly follows a characteristic progression from psychiatric disturbances to seizures, cognitive impairment, movement disorders, autonomic dysfunction, and reduced consciousness. Despite increasing awareness, diagnosis remains challenging because patients frequently initially present to psychiatric, acute medical, or cardiology services before the underlying autoimmune process is recognized [2,3,6].

Several aspects of this case warrant discussion. First, autonomic instability was a prominent and early feature of the illness. The patient experienced recurrent episodes of sinus tachycardia reaching 170-180 beats/min, prompting extensive cardiological investigations, including serial electrocardiography, echocardiography, troponin measurement, and assessment for pulmonary embolism. Anti-NMDAR encephalitis is known to cause autonomic instability through widespread disruption of central autonomic pathways and is generally associated with severe disease and intensive care admission. However, the presence of focal imaging abnormalities within the insular region, which is known to participate in cardiovascular regulation, makes this constellation of findings clinically interesting [2,3].

A particularly interesting feature was the presence of a focal right insular cortex abnormality on MRI. The insular cortex plays a critical role in autonomic regulation through its extensive connections with cortical, limbic, and brainstem autonomic networks. Lesions involving the insular region have been associated with cardiac rhythm disturbances, blood pressure variability, and autonomic imbalance. Nevertheless, the MRI abnormalities observed in the present case extended beyond the insular cortex itself, involving adjacent subcortical white matter [8]. Previous studies have demonstrated associations between insular dysfunction and cardiac rhythm disturbances, suggesting that inflammation involving this region may contribute to autonomic manifestations. Although causality cannot be established in an individual case, the coexistence of right insular cortex hyperintensity and significant sinus tachycardia raises the possibility that focal insular involvement contributed to the patient’s autonomic instability. This observation adds an interesting radiological-clinical correlation that may contribute to understanding autonomic manifestations in anti-NMDAR encephalitis [1,2]. Additionally, focal seizures themselves may provoke ictal tachycardia through activation of autonomic networks. Because continuous simultaneous EEG-cardiac monitoring was not available, it was not possible to determine whether episodes of tachycardia occurred independently of seizure activity or were temporally related to individual seizures.

Neuroimaging findings in anti-NMDAR encephalitis are often subtle or absent. Although MRI abnormalities can occur in approximately one-third to one-half of cases, findings are frequently nonspecific and may be erroneously attributed to seizure-related changes. In our patient, T2/FLAIR hyperintensity within the right insular cortex and adjacent subcortical white matter could initially have been interpreted solely as a postictal phenomenon. This case therefore reinforces the importance of interpreting MRI findings within the broader clinical context [1-3].

Electroencephalography provided additional supportive evidence of cerebral dysfunction. Although no epileptiform discharges or electrographic seizures were identified, the EEG demonstrated encephalopathic changes with excess right-predominant delta slowing. No extreme delta brush pattern was observed. These findings are consistent with previous studies demonstrating that EEG abnormalities are common in anti-NMDAR encephalitis but are often nonspecific. Although these observations suggest a possible relationship between the electrophysiological and radiological findings, the available data do not establish the localization of seizure onset or define the mechanism underlying the autonomic manifestations.

The diagnosis was supported by positive anti-NMDAR antibodies in both serum and CSF. Additional CSF analysis demonstrated mild lymphocytic pleocytosis and intrathecal IgG synthesis, as indicated by oligoclonal bands, further supporting an underlying neuroinflammatory process. Testing for alternative neuronal surface antibodies that could cause autoimmune encephalitis, including LGI1 and CASPR2, was negative [6].

This case also demonstrates the importance of escalation to second-line immunotherapy. Despite first-line immunotherapy, the patient continued to experience significant cognitive impairment and memory dysfunction. Given the incomplete response, rituximab was initiated following multidisciplinary discussion. Rituximab depletes CD20-positive B lymphocytes and has become an established second-line therapy for patients with persistent symptoms or an inadequate response to first-line treatment. The patient subsequently experienced progressive cognitive and functional recovery [4,6,7].

The patient underwent extensive pelvic imaging, which did not identify an ovarian teratoma or other neoplasm. Although ovarian teratomas remain the most well-recognized tumor association, particularly in adult women, a substantial proportion of patients have no detectable tumor. Tumor-negative anti-NMDAR encephalitis is increasingly recognized and should not delay the initiation of immunotherapy when clinical suspicion is high [2,3,5].

Finally, brief functional neurological symptoms emerged during recovery. Distinguishing these events from ongoing seizure activity was important to avoid unnecessary escalation of antiseizure therapy and demonstrates the complexity of neurological recovery after autoimmune encephalitis. The patient was followed up in the clinic and is now seizure-free and has returned to normal activities.

## Conclusions

Anti-NMDAR encephalitis should be considered in young adults presenting with new-onset psychiatric symptoms, focal seizures, cognitive impairment, and unexplained autonomic instability. This case highlights the coexistence of marked sinus tachycardia, focal seizures, right-predominant EEG slowing, and MRI abnormalities involving the right insular region and adjacent subcortical white matter in anti-NMDAR encephalitis. Although these findings raise the possibility of an interaction between autonomic dysfunction, focal cerebral involvement, and seizure activity, the available data do not establish a causal relationship. Sinus tachycardia may also represent a manifestation of the underlying encephalitic process and can lead to diagnostic distraction through extensive cardiac investigations. Early recognition, prompt immunotherapy, and timely escalation to rituximab in refractory cases may facilitate meaningful neurological recovery, even in the absence of an underlying ovarian teratoma.
